# Ptomaine Poisoning.—II

**Published:** 1911-08-26

**Authors:** H. J. Hutchens

**Affiliations:** Heath Professor of Comparative Pathology and Bacteriology in the University of Durham.


### Hospital Clinics.

### PTOMAINE POISONING.—II.

By H. J. HUTCHENS, D.S.O., D.P.H. Oxon., M.A.
Heath Professor of Comparative Pathology and Bacteriology in the University of Durham.

The various groups of intestinal bacilli can be readily differentiated by an elementary study of their bio-chemical reactions. The dysentery and typhoid bacilli give no gas in glucose or lactose, leave the colour of litmus milk unchanged, and form no clot. *B. paratyphoid* a gives gas in glucose, no gas in lactose, turns litmus milk permanently acid, but does not clot it. The enteritidis group gives gas in glucose, no gas in lactose, and turns milk distinctly alkaline. The colon group gives gas in glucose and in lactose, clots milk and turns it markedly acid. These differences may be summarised in the form of a table.

Table I.—*The bio-chemical reactions of the typhoid-colon group of bacilli, by means of which they can be differentiated.*

| | Glucose media. | Lactose media. | Litmus milk. |
|---|---|---|---|
| Dysentery bacilli (? mobile) ... | Acid, no gas | No change | Neutral, no clot |
| Typhoid bacillus | Acid, no gas | No change | Neutral, no clot |
| Para-typhoid bacillus ... | Acid and gas | No change | Acid, no clot |
| Enteritidis bacilli | Acid and gas | No change | Alkaline, no clot |
| Colon bacilli ... | Acid and gas | Acid and gas | Acid and clot |

The symptoms following infection with bacilli belonging to this group appear after a short incubation period (12 to 36 hours), and take the form of an acute gastro-enteritis accompanied in the more severe cases by vomiting, colic, and collapse. The temperature is generally subnormal, and from the clinical standpoint the disease in the severe cases is not unlike cholera. In one and the same epidemic the symptoms vary widely in their severity, some of those affected merely suffering from a slight diarrhœa, while in others an acute choleraic gastro-enteritis is followed by collapse and death. It often happens that a small percentage of those who partook of the infected food escape unharmed. But it is characteristic of these epidemics that by far the greater number who ate of the infected material are affected to a greater or lesser degree.

Poisoning by bacilli of the enteritidis group generally takes the form of a septicæmia. The bacilli can be recovered from the blood during life and from the spleen after death. But organisms of this group give rise to a highly thermostable toxin, which will resist boiling for some little time. This is a matter of considerable practical importance, because it can be readily understood that these organisms might be present in milk, for instance, in which they would multiply and produce their toxins. If the milk were heated before being used as food the bacilli might be destroyed, while the toxins remained, and these latter might then give rise to acute symptoms of gastro-enteritis in those who consumed it. This supposition is rendered all the more feasible by a consideration of animal experiments. Small laboratory animals fed on sterilised cultures of *B. Gärtner* or *B. Aertrycke* suffer from gastro-enteritis in the same way as though they had been fed with living cultures.

Infection with bacilli of the enteritidis group leads to the formation of agglutinins in the blood, and these are specific for the organism which was the cause of the infection. This fact is of practical importance, because in testing the blood of a person who is supposed to have suffered from food-poisoning due to organisms of this group it is obvious that blood will have to be tested as regards its agglutinating capacity, both for *B. Gärtner* and *B. Aertrycke*. It is also important to remember that co-agglutinins also appear, and unless care be taken to bear this fact in mind a Gärtner infection might be mistaken for an enteric infection when the agglutination reaction alone is relied upon, because of the considerable amount of co-agglutinin for the typhoid bacillus which is often found in a Gärtner serum.

Epidemics due to *B. Gärtner* have been recorded by Gärtner at Frankenhausen, by van Ermengen at Morseele, Brussels, and Gand, by McWeeney at Limerick, etc. In the neighbourhood of Newcastle recently, symptoms of enteritis affecting more than 100 persons were traced to the consumption of milk from a particular farm. The farm was visited, and it was found that a cow, which had recently calved, was suffering from enteritis. The cow died, and post-mortem examination "revealed tuberculous pleurisy, while the kidneys and liver were completely disorganised and the fore stomach inflamed." None of the cases proved fatal. *B. Gärtner* was isolated from the stools of some of the patients, and was present in practically pure culture in the milk. Unfortunately, none of the tissues or organs of the cow could be obtained for bacteriological examination.

The *Bacillus enteritidis* (Aertrycke) has been isolated from epidemics at Aertrycke by de Nobelé, at Breslau by Kœnsche (this organism was originally described as *B. Breslaviensis*), at Düsseldorf by Trautmann, at Neunkirchen by Drigalski, etc., and it was proved to be the cause of an epidemic at Chadderton by Durham. In Newcastle in December last, twenty-one persons ate some tinned mutton; nineteen became ill with symptoms more or less severe of gastro-enteritis, and one case proved fatal. *B. Aertrycke* was isolated from the spleen and intestinal contents of the fatal case and from the mutton.

The mortality in epidemics of food-poisoning due to bacilli of the enteritidis group is somewhat variable, but on the whole low. At Frankenhausen (Gärtner) 58 persons were ill and one died; at Cotta (Gärtner) 136 were ill, four died; at Morseele (van Ermengen) 4 out of 80 affected died; at Breslau (Kœnsche) 80 became ill, none died; at Derby

(Delépine) 210 at least were affected, 4 died; at Neunkirchen (Drigalski) 30 were ill, 3 died; at Dusseldorf (Trautmann) 57 were affected, 1 died; at Limerick (McWeeney) 93 affected, 8 died; at Newcastle (Hutchens), in the milk epidemic at least 105 were ill and none died, in the mutton epidemic 19 were affected and 1 died.

From the point of view of prophylaxis, great interest naturally attaches to the source of these organisms. So far as evidence is available it would seem that the lower animals, cattle, pigs, and horses, suffer from an acute specific infective septicæmia, accompanied by enteritis, and due to bacilli of the enteritidis group, and in the great majority of cases some history of disease in the animal whose flesh was the cause of an epidemic of food-poisoning can be obtained on careful inquiry. Van Ermengen has collected details of 112 epidemics of meat poisoning. In 103 of these epidemics the meat came from a diseased animal. In the milk epidemic already referred to one of the cows at the farm from which the milk came was suffering from some acute disease accompanied by diarrhœa. As a rule, however, the meat has a perfectly healthy appearance, and is quite above suspicion. In van Ermengen's series, quoted above, out of the 103 epidemics in which the meat was traced to a diseased animal in only five did it show evidence of putrefactive change. Experiments have been carried out to ascertain if organisms of the enteritidis group are normally present in the healthy human and animal intestine, and some German observers have stated that these bacilli are to be found fairly frequently under such conditions, Savage in a long series of careful investigations has failed altogether to confirm these results, and expresses the view that food-poisoning in man is an infection derived from animals actually suffering from disease caused by bacilli of the enteritidis group.

Organisms having the morphological and cultural characters of the enteritidis group have been obtained from numerous sources, and have received different names. In addition to the *B. enteritidis* (Gärtner) and *B. enteritidis* (Aertrycke) which have already been mentioned, the following organisms, among others, all belong to this group:—

1. An organism which has been found in association with a disease clinically resembling enteric fever in man, and known as the *bacillus paratyphoid β.*
2. An organism isolated from pigs suffering from hog-cholera, and known as the bacillus of hog-cholera, *B. suipestifer*, etc.
3. The *Bacillus typhimurium* (Löffler).
4. The bacillus of Danysz, found in a disease of rats.
5. *Bacillus psittacosis*, isolated by Nocard from parrots suffering from a septicæmic condition, accompanied by enteritis and congestion of the internal organs.

It has already been pointed out that while *B. Gärtner* and *B. Aertrycke* cannot be distinguished on morphological or bio-chemical grounds, they can, however, be readily differentiated by agglutination reactions. Both these organisms were originally isolated from typical epidemics of food-poisoning, and have since been found in numerous similar epidemics, and neither has ever been found except in association with epidemics of food-poisoning. Clinically the symptoms produced by them are the same, and whether an epidemic is due to *B. Gärtner* or *B. Aertrycke* can only be determined by a study of the agglutination reactions of the organism isolated. From some cases, however, of disease in man clinically indistinguishable from enteric fever, an organism, *B. paratyphoid β*, has been isolated, which, so far as its morphological and bio-chemical reactions are concerned, is identical with *B. Aertrycke* and *B. Gärtner*. Boycott, investigating 176 cases of disease in man which had the clinical symptoms of enteric fever, proved that three of them were infections with *B. paratyphoid β* and showed that two others were probably of the same nature. At the same time epidemics of food-poisoning have been recorded as due to *B. paratyphoid β.* Hence a difficulty has arisen as to the exact rôle which *B. paratyphoid β* plays in human pathology. Trautmann expressed the opinion that from the point of view of ætiology food-poisoning and paratyphoid β fever are different manifestations of one and the same disease, the former being an acute attack (toxins and micro-organisms), the latter a sub-acute attack (micro-organisms alone). Boycott, however, does not appear to regard this explanation as satisfactory. In his opinion "the morbific relations to man are different, for while hog-cholera, Aertrycke, etc. gives rise to a sudden acute illness (food-poisoning), paratyphoid β causes a disease with no clear clinical distinction from enteric fever." With a view to determining the relationship of *B. paratyphoid β* to the other members of the enteritidis group, Bainbridge has made an elaborate study of the agglutination and absorption reactions of these organisms, and has been able to show that the enteritidis group comprises three organisms, *B. Gärtner*, *B. Aertrycke*, and *B. paratyphoid β.* The first can be distinguished from the two latter by agglutination reactions, but the two latter can only be differentiated by a study of their absorption phenomena, as they both show the same agglutination reactions with the same serums. These results obtained by Bainbridge have been confirmed by Dean after an investigation of the complement deviation reactions. These researches would at first sight appear to offer an explanation of the difficulty with regard to the rôle of *B. paratyphoid β.* For it would seem quite likely that hitherto *B. Aertrycke* and *B. paratyphoid β* have been confused, and that "until the bacilli isolated in outbreaks of food-poisoning have been examined by this [absorption] method (in addition to their cultural and agglutination reactions) the possibility that *B. paratyphoid β* can give rise to the usual symptoms of food-poisoning remains unproved. Meanwhile, so far as can be judged from the available bacteriological evidence, the distinction drawn on clinical grounds between infection by *B. Aertrycke* and by *B. paratyphoid β* still holds good" (Bainbridge). Since this was written, however, Bainbridge has recorded a small epidemic of food-poisoning due to *B. paratyphoid β*, so that the difficulty cannot be said to have disappeared, and further observations along the lines indicated by Bainbridge are necessary.

(*To be continued.*)

